# Hair Cortisol in Sheltered Cows and Its Association with Other Welfare Indicators

**DOI:** 10.3390/ani9050248

**Published:** 2019-05-16

**Authors:** Arvind Sharma, Govindhaswamy Umapathy, Vinod Kumar, Clive J. C. Phillips

**Affiliations:** 1 Centre for Animal Welfare and Ethics, School of Veterinary Science, The University of Queensland, Gatton Campus, Gatton QLD 4343, Australia; c.phillips@uq.edu.au; 2 Laboratory for the Conservation of Endangered Species, Centre for Cellular and Molecular Biology, Hyderabad 500007, India; guma@ccmb.res.in (G.U.); vinod@ccmb.res.in (V.K.)

**Keywords:** hair cortisol, cows, shelters, welfare, measures, resources, indicators

## Abstract

**Simple Summary:**

Hair cortisol concentrations are measured for the assessment of long-term stress in animals. The objective of this study was to assess the levels of stress in retired, abandoned and unproductive cows housed in traditional shelters through the measurement of their hair cortisol levels. The study further aimed to explore the association of the hair cortisol concentrations with other cow and resource-based welfare indicators. High hair cortisol levels were associated with dung lying in the sheds, low dry bulb temperature and shelters having little access to the yards. At the cow level, high hair cortisol levels were associated with injuries on the joints and body, dehydration, old age, and low body hair loss level. The study concluded that hair cortisol is an effective tool to assess stress levels in cows under field conditions.

**Abstract:**

India, the country with the largest population of dairy cows in the world, has a policy of retiring abandoned and non-lactating cows in shelters, but the level of provision for their welfare in these shelters is unclear. Cows in 54 shelters across India were assessed for historic evidence of physiological stress, through determination of hair cortisol in 540 samples from 10 cows in each shelter by enzyme immunoassay. Animal-based and shelter resource-based welfare measures were recorded and correlations with the hair cortisol investigated by multivariable analysis. High hair cortisol concentrations were associated with dung in the lying area of the cowshed, a low dry bulb temperature there and little cow access to yards, as shelter-based variables. At a cow level, high hair cortisol concentrations were associated with dirty flanks, hock joint ulceration, carpal joint injuries, body lesions, dehydration, an empty rumen, old age, and low levels of body hair loss. Hair cortisol level promises to be an effective biomarker of stress in cows when conducting studies under field conditions.

## 1. Introduction

Hair cortisol is a biomarker of chronic stress in animals and its analysis provides an objective assessment of hypothalamic pituitary adrenal (HPA) axis activity over a long time period [1]. As a welfare measure, it is non-invasive, valuable for longitudinal studies, has a long-time lag for changes and is especially useful for field studies [2,3,4,5,6,7,8,9,10,11]. The other matrices for detection of cortisol, principally urine, blood, saliva and faeces, cannot provide long term retrospective evaluations of cortisol [12,13,14]. Hair cortisol analysis is also more reliable to assess long term stress than blood, saliva, urine and faeces because the sebum of hair has lipophilic properties, which facilitate the effective binding and aggregation of the circulating cortisol in the shafts [1,5,11,13,15]. Hair analysis is now being used to detect long-term retrospective levels of cortisol in farm animals, principally cattle [9,13,15,16,17,18,19]. It has also been analysed in humans [12], dogs [20], horses [21], pigs [22] and wild animals, such as rhesus macaques [10], polar bears [4], rats [23], coyotes [24], and kangaroos [25] for studying reproductive and adrenal endocrinology.

Studies have demonstrated the sensitivity of hair cortisol in cattle to the stresses of changes from winter indoor housing to summer pasture grazing and changes in nutrition [15,16]. Enzyme Immunoassay (EIA), Enzyme-Linked Immunosorbent Assay (ELISA), and Radioimmunoassay (RIA) techniques have been deployed to detect and validate milk, plasma and hair cortisol concentrations in cows [16,17,18,19,26,27]. However, there is a paucity of information relating to hair cortisol with other welfare indicators for cattle. The purpose of this study was, therefore, to assess hair cortisol concentrations in a range of old, retired and unproductive cows housed in traditional cow shelters or retirement homes (gaushalas) in India and explore its association with other indicators of welfare, measured both on the cows and in their housing conditions. This study was a part of a larger study of the welfare assessment of cows in the cow shelters.

## 2. Materials and Methods

This research study was conducted with animal ethics and human ethics approval from the University of Queensland Animal Ethics Committee (approval number SVS/CAWE/314/16/INDIA). A sample size of 54 shelters was selected based on a power analysis [28] which indicated that a sample size of 50 shelters would be an adequate representation of shelters in major Indian states. Hence a total of 54 cow shelters were selected in six states of India (Gujarat, Maharashtra, Rajasthan, Punjab, Haryana and Himachal Pradesh. The study was conducted from December 2016 to July 2017. The criteria for selecting a shelter were: a minimum of 30 cows, that it was not a commercial dairy unit (defined as a shelter not selling more than 20 litres milk/day), and that the shelter was managed by a government, temple, public or a philanthropic trust. We then performed power calculations based on a review of published hair cortisol studies [13,15,16,17,18,19,27,29] that suggested a mean hair cortisol concentration with standard error estimates of 4.99 pg/mg and standard deviation of ±3.65 pg/mg. To detect a 10% difference between our samples and a reference sample added to our samples at a *p*-value of 0.05 and a power of 0.8, we determined that the sample size should be 419 cows [28]. In each shelter, 10 cows that were confirmed by the manager and shelter records had been in the shelter at least 6 months were selected randomly by choosing every third cow in the shed or the yard until the sample size was attained.

### 2.1. Welfare Measurement

These cows were further assessed for their welfare in the shelters by the measurement of both cow and shelter-based parameters. One of the authors (AS) undertook a two-day course on low stress livestock handling and a three-month training in scoring the cows for assessment of body condition, lameness, claw overgrowth avoidance distance, dirtiness, limb lesions (joint hair loss, ulceration and swellings), skin lesions, rumen fill, faecal consistency and rising behaviour, at the School of Veterinary Science, The University of Queensland. Pilot trials were also conducted to validate the selected welfare measures in two shelters before the commencement of the actual data collection. One of the researchers (AS), a veterinarian, led all the assessments in the shelters. The cow-based welfare parameters (Appendix A) assessed were as follows: lactation status (lactating or non-lactating), Body Condition Score (BCS) on a scale of 1 to 5 [30,31], in increments of 0.25, with score ≤1.25 indicating emaciation, 1.5–2 indicating thin, 2.25–3.75 normal and 4 or more obese. General demeanour was assessed by modifying a five-point scale formulated by Cafe et al. [32] into a dichotomized scale, docile or aggressive.

#### 2.1.1. Cleanliness, Lesions and Disease Measures

Details of individual scoring systems are presented in Table A1 (in Appendix A). Dirtiness of the hind limbs, udder and flank and body hair loss were scored as described by Whay et al. [33]; swellings, hair loss and ulceration of the hock joints and carpal joint injuries using the four-point scales of Wechsler et al. and Whay et al. [33,34]. Lesions were presumed to be predominantly acquired from shelter furniture as a consequence of interaction with sharp nails/metals protruding from shelter gates and/or barbed wire fencing, and manifested in the form of hair and tissue loss. Sharp lacerations and avulsion of the skin were described using the method of Huxley and Whay [35], neck lesions by the method of Kielland et al. [36] and ocular lesions, nasal discharge, hampered respiration, diarrhoea and vulvar discharge by the method of Coignard et al. [37]. Rumen fill score and the consistency of faeces was evaluated according to the method of Zaaijer and Noordhuizen [38] and lameness was assessed using the locomotion scores referred to by Flower and Weary [39]. Claw overgrowth was visually assessed using the scale devised by Huxley and Whay [35]. Skin lesions or integument alterations were evaluated using the method of Leeb et al. [40].

We formulated our own protocols for teat and udder scoring, skin tenting time, to assess dehydration, and the presence of oral lesions, because it was anticipated that emaciation, teat and udder abnormalities and the presence of very old cows would be more common in the shelters than in dairy cow farms, for which other scales had been developed.

Ectoparasitism was scored using a modification of the method devised by Popescu et al. [41].

#### 2.1.2. Cow Behaviour Measures

The avoidance distance (AD) of the sampled cows in each shelter was used as recommended in the Welfare Quality^®^ protocol [42]. A cow was approached from immediately in front at a rate of one step per second, starting at 2 m from the manger. The distance between the assessor’s hand and the cow’s head was estimated at the moment the cow moved away and turned its head, using the following four categories (Table A1). Rising difficulty of a sample of 10 cows that were lying down in each shelter was categorized using an existing protocol [43,44]. All the cows lying in the shelter were coaxed to get up with the use of a minimum amount of force. If the presence of the assessor did not evoke rising they were given one or two moderate slaps on the back, followed by more forceful ones if necessary (for four cows only).

#### 2.1.3. Shelter-Based Measures

Shelter-based resource assessments were based on housing features, including cleanliness, bedding, flooring, and water and feed provisions in the shelter. First, the total number of sheds per shelter and the number of animals per shed in the shelter was assessed, then two representative sheds were selected if more than two were present. Then the length, breadth and height of the sheds were recorded using a laser distance meter (CP-3007 model, Ultrasonic distance meter 40 KHz frequency, Chullora, New South Wales, Australia) and confirmed for each one using a measuring tape. From these measurements, the area of the shed and area per cow were calculated. The space allowance per cow in shelters having loose housing was calculated by dividing the floor area of the stall by the total number of cows within. In shelters with stalls, the area/cow was calculated from the floor area of each stall housing a cow [45,46]. In tethered stalls, the area per cow was calculated by measuring the distance from the end of the rope at the point of attachment to a peg to the end of the hind limb of the cow at full extension. This length was used as a radius to calculate the maximum potential area of movement of the tethered cows in the sheds.

Luminosity in the sheds was measured using a light meter (LCD Digital Lux Light Meter 9V Tester LX1010B 0 with 100,000 FC Photo Camera, Shenzhen Yongxiang Science and Technology Co., Ltd., Shenzhen, China) pointed in all six possible directions of the face of a cube at the centre of the shed. The mean of the six readings was calculated for each shelter. Dry and wet bulb temperatures were recorded using a digital meter (TS-FT0423 Digital Wireless Indoor Outdoor Thermo-Hygrometer Thermometer Humidity Meter, Sydney, Australia) inside the shelters before any cows were removed. The gradient of the floors in the sheds and the yards were measured at three different places as vertical and horizontal measurements with an inclinometer (Bosch Professional, 600MM, DNM60L Model, Bairnsdale Electrics, Victoria, Australia). Noise levels in the cow shelters were measured at three different locations in the sheds and yards within the herd using an android phone application [47]. Friction levels of the shelter floors were determined as the Coefficient of Friction (CoF), the force required to move an object over a floor divided by the weight of that object [48,49]. This was estimated using a 1 kg/10 N spring balance attached by a hook to a cuboid wooden block (mass 156 g). The block was gently pulled across the floor at a speed of 0.17 m/s and the minimal frictional force (F) required to keep it moving was recorded [50].

The type of housing (free stall, tie stall, loose, tethered or no housing); roofing (portal, flat, sloped or other); and shed flooring (brick, stone, earthen, concrete or other); presence of bedding in the sheds (present or absent); type of bedding if present (hay, straw, rubber mats or other) and the presence of yards (present or absent) and number of trees in the shelter yards [46,51,52,53], watering provisions and the number and types of water points (troughs, bowls, natural water bodies or other), were recorded in all the selected sheds and/or yards [45,52]. The cleanliness of the shelter premises was recorded by visually assessing the mean percentage of the floor that was covered by dung and urine in the sheds, passages and the yards separately [54]. The information about the duration of cows’ access to these yards (in h/day); access to pasture grazing (present or absent) and duration of access to the pastures (in h/day) was obtained from the interview of the shelter manager.

### 2.2. Hair Cortisol

#### 2.2.1. Sampling

Hair samples of approximately 5 g were taken in triplicate from the switch of the tail only, cutting from the base at skin level using scissors disinfected with 70% alcohol between cows, a site recommended in a previous study [27] for hair cortisol analysis, and stored in individual plastic ziplockbags at room temperature (approximately 20 °C) in the dark before processing. Hairs present at the switch of the tail were collected irrespective of their colour.

#### 2.2.2. Extraction of Cortisol from Hair

Cortisol was extracted from hair samples using a protocol described by Davenport et al. [10] and modified by Tallo-Parra et al. [13]. Approximately, 250 mg of hair was weighed and washed with 5 mL of isopropanol to remove the external steroid sources. The hair samples were washed twice with water and twice with isopropanol for 3 min each wash to remove the external steroids and dirt. Approximately 250 mg of hair sample was placed in a 15 mL falcon tube before adding 5 mL of water and vortexed for 3 min at room temperature. The samples were then dried, adding 5 mL of Isopropanol and vortexing for 3 min at room temperature to remove the excessive dirt, urine and faecal contamination. The hair sample was then allowed to dry for 3–4 days in a hot air oven at 40 °C, after which it was minced into 2 mm lengths and pulverized manually into a fine powder using a pestle and mortar. Then 50 mg of hair powder was weighed into 2 mL micro centrifuge tubes, 1.5 mL of absolute methanol was added and shaken at 100 rpm for 18 h at 30 °C for extraction of steroids. After incubation, tubes were centrifuged at 7000× *g* for 2 min. Following centrifugation, 0.75 mL of supernatant was transferred into a fresh vial and kept in an oven at 38 °C for drying the supernatant for 24 h. Dried extracts were reconstituted with 300 µL of EIA assay buffer (0.1 M PBS, pH 7, containing 0.1% BSA), vortexed for 30 s and stored at −20 °C until analysis.

#### 2.2.3. Cortisol Enzyme Immunoassay (EIA) for Determination of Hair Cortisol Concentration

**Figure 1:**
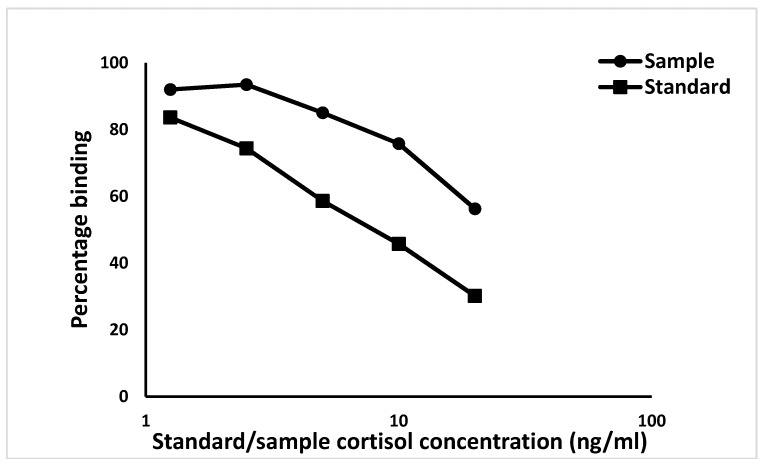
The parallelism between the serial dilution of pooled hair extracts of cow samples and cortisol standards.

Hair cortisol samples were analysed at the Centre for Cellular and Molecular Biology in the Laboratory for the Conservation of Endangered Species, an internationally recognized endocrinology laboratory, by three of the authors (G.U., V.K. and A.S.). The hair cortisol concentrations were measured using a polyclonal cortisol antibody (R4866, provided by Dr. Coralie Munro, University of California, Davis, CA, USA), diluted to 1:9000 in the assay. Cross-reactivity of polyclonal cortisol antibody approximated 100% with cortisol, prednisolone 9.9%, prednisone 6.3%, cortisone 5% and <1% with corticosterone, desoxycorticosterone, 21-deoxycortisol, testosterone, androstenedione, androsterone and 11-deoxycortisol [55,56,57]. The cortisol antibody sensitivity was calculated at 90% binding and found to be 1.95 ng/well. The inter- and intra-assay coefficients of variation (CV) of the assays were 7.19% (*n* = 10) and 2.68% (*n* = 10), respectively. Hair extracts were pooled and serially diluted (1:2, 1:4, 1:8, 1:16, 1:32) in triplicates (three repetitions i.e., each dilution was made in triplicates) to determine the parallel displacement curves between the pooled hair extract and respective standard of cortisol. Parallelism is the way to determine the immunological activity of antigen (cortisol in hair extract) and antibody (cortisol antibody) using serial dilutions at 50% binding. Parallel displacement curves were drawn to determine the relationship between the pooled serial dilution of hair extracts and their respective standards [57] (Figure 1). The enzyme immunoassay (EIA) was performed using the previously described procedure [55,56,57].

### 2.3. Statistical Analyses

Statistical analyses were performed using the software Minitab 17 Statistical Software (Minitab^®^ version 17.1.0, Minitab Ltd., Pennsylvania State University, State College, PA, USA), with the data presented in Table S1, following removal of outliers. Prior to statistical analysis, all data were tested for normal distribution by means of Anderson–Darling test and visualisation of probability distribution curves. Descriptive statistics were calculated and expressed as median, first quartile (Q1), third quartile (Q3) and interquartile range (IQR), as the data were not normally distributed. A univariate analysis was done to evaluate the relationships between various analysed parameters by performing the Spearman’s Rank Correlations for cow-based welfare parameters and shelter-based parameters separately. The statistical significance was set at *p* ≤ 0.05. Then a multivariable analysis was undertaken to reveal associations between the cow hair cortisol (response variable) and other cow-based parameters at the individual cow level as well as with the shelter-based welfare parameters. A principal component analysis was performed in both cases to reduce the data and avoid multicollinearity in order to explain the maximum variance with least number of principal components. The variables which were omitted were lesions from shelter furniture, vulval discharge, neck lesions and hampered respiration. The principal components with eigenvalues of more than one were considered for entry into a stepwise General Linear Model with alpha to remove variables of 0.05. The final models were evaluated for validity by taking into account adjusted r^2^ and *p*-values of the factors and the independency of factor variables assessed by variance inflation factor (VIF) statistics. Factors with VIF < 10 were considered to show the absence of multicollinearity between factors. The assumptions of homoscedasticity and normal distribution of the residuals were tested graphically. Stability of the modelling process was evaluated by comparing the models from forward and backward selection methods.

## 3. Results

### 3.1. Animal and Shelter Based Measures

The median hair cortisol concentration was 1.43 pg/mg (IQR = 1.02 pg/mg). Descriptive statistics for animal-based and shelter-based parameters are shown in Table 1 and Table 2. None of the parameters, animal- or shelter-based were normally distributed. Out of the 540 sampled cows, median age was 11 years; most were non-lactating, docile, of intermediate body condition and had mild to moderate dirtiness of the hind limbs, udder and flanks. Most had no or only a mild hair loss, mild to moderate hock joint swelling and hair loss on their hock joints, but no, or only mild carpal joint injuries (swelling, hair loss and ulceration). Few cows had lesions on their necks or bodies. There was some evidence of nasal discharge, lameness, claw overgrowth, teat, udder and ocular lesions but little evidence of diarrhoea. Rumen fill was usually intermediate. Mild to moderate levels of ectoparasitism were recorded, mainly in the form of lice and tick infestation, but there was little evidence of clinical dehydration, as evidenced by a skin tenting time. The avoidance distance scores indicated an ability to approach the cows to close range and mostly had had a normal sequence of rising.

The median number of cows per shed was 70, and the shed area per cow was 2.73 m^2^. The median percentage of dung in the lying areas and passages of the sheds was 15% and 10%, respectively. In 83.3% of the sheds (45 sheds) and 88.8% of the yards (48 yards), there was no accumulation of urine in the lying areas and passages. There was no provision of bedding in 96.3% (52 shelters) of the shelters; only two shelters had paddy straw bedding, 0.03 and 0.05 cm thick. There was no water run-off in the lying areas in 72.2% of the shelters (39 shelters). The median height of the eaves of the shed roofing was 3.80 m. The median gradients of the shed flooring in the lying areas and passages was 1.46 and 2.36, respectively, and median CoF of shed floors 0.43. The median luminosity and noise levels in the shed were 582 lux and 27.7 decibels, respectively. The median dry and wet bulb readings in sheds were 29.5 °C and 34%, respectively. There was only one water point in 48% of the shelters, which was mostly located in the yards. Water points were absent in the sheds in 71% shelters. Twenty-three per cent of the shelters had no water points in the yards, 48% had one water point, 18.5% had two water points and only 10.5% shelters had three or more (up to six) water points in their yards. A median 20% of the floor was covered with dung in the shelter yards, but most shelters (88.8%, *n* = 48) had no urine on the yard floors.

A median of 8 h of access to the yards was provided to the cows in the shelters. The median yard area per cow was 5.9 m^2^ and the median CoF and gradient of yard flooring were 0.64 and 1.51. The median noise level in the yards was 25.3 decibels, and the median number of trees in the yards was 2. There was no access to pastures for the cows in 59.2% of the shelters (32 shelters), and 26% of the shelters (14 shelters) provided access to pastures for up to 6 h/day. The median frequency of feeding the cows was three times a day, with the median quantity of fodder fed on a daily basis being 17.5 kg. Dry straw was fed in 18.3% shelters (*n* = 10), dry straw with agricultural by product waste in 20.4% (*n* = 11), dry straw with agricultural by product waste and hay in 46.4% (*n* = 25) and all the three along with greens and vegetable waste in 14.9% shelters (*n* = 8). Though 86% of the shelters provided concentrates in the form of rice or wheat husk and grains, the quantity received by each cow was less than 0.5 kg/day.

### 3.2. Correlations between Hair Cortisol and Animal and Shelter Based Measures

Several animal-based measures showed weak but significant correlations with hair cortisol (Table 3). At the shelter level, the Spearman’s Rank Correlations detected a significant positive correlation (CC = −0.298, *p* = 0.028) between hair cortisol concentration and the presence of runoff water in the shed lying areas. A significant negative correlation (CC = −0.370, *p* = 0.006) indicated that the hair cortisol concentration decreased with increasing duration of access of the cows into the yards and with the cleaning of areas other than sheds and yards (CC = −0.317, *p* = 0.019). Significant correlations were observed between variables in both animal and shelter based measures (Table 4 and Table 5). The correlation matrix for the all the variables in animal-based and resource-based measures is depicted in Table A2 and Table A3 (in Appendix A).

The multivariable analysis of the animal-based measures with hair cortisol revealed positive correlations with: dirty flanks, hock joint ulceration, carpal joint injuries, lesions on the body skin tenting time, age of the cows and lactation status and a negative correlation with body hair loss and rumen fill score (Table 6). The total r^2^ adjusted was 20.98% and residuals were normally distributed.

The relationship was described by the equation:

Hair Cortisol Concentration (log_10_pg/mg) = c + 0.20 (±0.084, *p* = 0.017) + 0.07 Dirty flanks score (±0.0142, *p* < 0.001) − 0.06 Body hair loss score (±0.0180, *p* = 0.001) + 0.03 Hock joint ulceration score (±0.0150, *p* = 0.04) + 0.04 Carpal joint injuries score (±0.0139, *p* = 0.002) − 0.06 Rumen fill score (±0.0195, *p* = 0.002) + 0.036 Lesions on the body score (±0.0182, *p* = 0.04) + 0.08 Skin tenting time score (±0.0252, *p* < 0.001) + 0.0058 Age of the cows (±0.0028, *p* = 0.03), (1)

where c is the intercept, which was 0.236 for non-lactating cows and 0.165 for lactating cows (*p* = 0.02); r^2^ adjusted = 20.98%; residuals were normally distributed.

The multivariable analysis of the shelter-based measures with the mean hair cortisol concentration in cows at the shelter level produced a positive correlation between hair cortisol concentration and % dung in the lying area of the cowshed, and negative correlations with dry bulb temperature reading in the shed and the duration of access of the cows to the yards (Table 7). The relationship is described by the equation:

Hair cortisol concentration = c + 0.016 Percentage of dung in the lying area of the cowshed (±0.00597, *p* = 0.02) − 0.15 Dry bulb reading in the shed (±0.0298, *p* = 0.001) − 0.070 Duration of access to the yard (±0.0241, *p* = 0.01), (2)

where c is the intercept, which is 6.15 (*p* < 0.001); r^2^ adjusted = 65.69%; residuals of the model were normally distributed following visual inspection of their graphical representation.

## 4. Discussion

### 4.1. Hair Cortisol Concentrations

The hair cortisol concentration in our study was in the similar range to that recorded in some studies in dairy and beef cattle [13,15,16,17,18,27,29]. Though the median hair cortisol concentration was lower in our study, it was still within the similar range reported in previous studies (Table 8). The hair samples in our study were cut into 2 mm pieces and pulverised manually, as recommended to maximise extraction of hair cortisol [13,18]. A major difference between our study cows and those cited above was that we had a much larger number of cows, over a wider geographical area with different agro-climatic conditions and management practices. The different analysis protocols, extraction procedures, climatic and breed variabilities are important factors affecting the results of hair cortisol estimation. There is interplate and intraplate variation in the estimation process, which was below 6% in this study. This is acceptable, and each plate sample was mixed for the required period of time.

### 4.2. Hair Cortisol and Animal-Based Measures

The low hair cortisol concentration in the cows with hair loss is in contrast to the findings of Novak et al. [58], who observed a positive correlation between hair loss and hair cortisol concentration in Rhesus Macaques. However, this study was inconclusive on whether the relationship between hair loss and hair cortisol concentration was causal or just an association. Moreover, one of the sub groups of macaques showed no relationship between hair loss and elevated hair cortisol concentrations. There is a “wash out effect,” in which there is a decline in the hair cortisol concentration from the proximal segments to the distal ones [59] due to the ultraviolet radiation [60] or due to the effect of grooming and licking in animals [61]. The most plausible reason for our result is adrenal gland fatigue due to extended periods of overactive cortisol production. The overworked adrenal gland works less efficiently and might lead to less cortisol production and other glucocorticoids, which may lead to hair loss. Studies in humans have shown that subjects with hair loss express reduced levels of glucocorticoids due to a weak response to stress [62,63]. However, the adrenal gland fatigue theory has been rejected in a systemic review by endocrinologists [64] citing the absence of substantive proof of this condition due to the methodological and confounding errors in various studies on the relationship between HPA axis activation and adrenal gland fatigue. The cows in the shelters suffer chronic stress due to the health and managemental issues such as old age, low quality feeding practices, less area/cow, improper flooring and cleanliness, highlighted in this study which could activate the HPA axis leading to elevated hair cortisol concentrations. This is a cross sectional study at a point of time which might not fully explain the causality of the elevated hair cortisol concentrations in shelter cows. A prospective study is recommended to further explore this relation between the HPA axis activation and adrenal gland fatigue.

The positive association between the dirtiness of the flanks and hair cortisol in the shelter cows may derive from an indirect effect of dirtiness on stress levels in the body, as dirtiness predisposes animals to diseases and injuries [65]. Dirtiness reduces hygiene of the cows and exposes the risk of pathogens leading to disease which causes stress [66,67]. The dirtiness of the animals could be due to improper management and high stocking density in the housing facilities [68]. The matting of the hair caused by dirtiness might cause minor haemorrhages, putting tension on the epithelial tissue of the skin when strained leading to pain and stress [69]. Faecal contamination of the cows’ hair coat causes discomfort, reduces thermoregulation and increases the incidence of disease [70]. The area per cow in our study was much lower than the recommended for comfort [48,71] which might have led to dirtiness and stress, thus accounting for the positive correlation between dirtiness of flanks and elevated hair cortisol concentrations. Significant univariable positive correlations were observed between dirty flanks and body hair loss (CC = 0.42, *p* ≤ 0.001), carpal joint injuries (CC = 0.33, *p* ≤ 0.001), lesions on the body (CC = 0.33, *p* ≤ 0.001), ectoparasitism (CC = 0.22, *p* ≤ 0.001), diarrhoea (CC = 0.14, *p* = 0.002) and skin tenting time (CC = 0.25, *p* ≤ 0.001). A negative correlation was observed between dirty flanks and rumen fill score (CC = −0.22, *p* ≤ 0.001). The positive univariate relationships reveal that the interplay of these animal health indicators is correlated with changes in the hair cortisol concentration in the shelter cows. The effect of dirtiness on the health of cows has been documented in previous studies, underlying the importance of cleanliness in reducing health risks [67,72]. The associations between cleanliness and lesions on the joints and integument alterations have also been reported [73].

Hock joint ulceration at the tuber calcis, carpal joint injuries and lesions on the body are painful traumatic lesions which lead to inflammation. The positive correlation between the hair cortisol concentration and the carpal joint injury score and body lesions’ score is probably attributable to the activation of the HPA axis due to the stress response of the body to these injuries, at least in dairy cattle [18]. However, in our study, the hair cortisol concentration was found to be elevated in sub clinical health problems (joint and skin injuries and swellings) in contrast to the findings of Burnett et al. [18], who found no elevation in sub clinical endometritis. This could be because of greater stress caused by the injuries in the limbs and joints than in the case of endometritis.

The negative correlation between rumen fill score and hair cortisol concentration, though weak, may justify its inclusion in the welfare assessment protocol as a cow health signal [74], being indicative of dry matter intake, fluid intake, the composition of feed, digestibility and the passage rate of the ingested feed [38,75,76,77]. Almost 60% of the cows in our study had a score of 4 which shows low fluid intake and more dry matter, as is common for dry cows. Rumen fill score also indirectly provides an indication of underlying sub clinical disease due to changes in feed intake or dry matter intake [78]. Rumen fill score indirectly provided information about the feeding management, and the latter could be a potential stressor in the shelter cows. Rumen fill score has been used as an indicator of poor health and nutritional stress in cows [79]. In this study it provides information about the lack of balanced nutrition and health of the cows due to its significant negative univariable association with diarrhoea (CC = −0.12, *p* = 0.006), ocular lesions (CC = 0.18, *p* ≤ 0.001), hock joint ulceration (CC = −0.15, *p* ≤ 0.001), carpal joint injuries (CC = −0.14, *p* = 0.001), lesions on the body (CC = −0.32, *p* ≤ 0.001), lameness (CC = −0.12, *p* = 0.006) and claw overgrowth (CC = −0.18, *p* ≤ 0.001), as previously reported (Table A2 and Table A3) [50]. Most of these lesions induce chronic pain and could potentiate stress in the cows depicted by elevated hair cortisol levels. The association of rumen fill score with these other health parameters in this study should be interpreted with caution as these scores change over a 24 h period and in a cross-sectional study at a point of time does not indicate a causal relationship. A routine measurement of this parameter in a cow herd has been suggested to interpret its relevance to predict the cows at risk of developing disorders [80].

Age and lactation showed a positive association with hair cortisol concentration and are in agreement with Burnett et al. [18]. Lactating cows are challenged physically, metabolically and immunologically as a result of production stress, clinical and sub clinical diseases and immune suppression [81]. Aged cows are normally multiparous and harbour subclinical health disorders like metritis that might activate the HPA axis through inflammatory conditions [82], even though Burnett et al. [18] did not find that sub clinical conditions of endometritis increased hair cortisol. Lactation had significant positive correlations with BCS (CC = 0.15, *p* = 0.001) and coat condition (CC = 0.12, *p* = 0.004) in our study. Contrarily, significant negative relationships between lactation and teat and udder score (CC = −0.59, *p* ≤ 0.001), ectoparasitism (CC = −0.14, *p* = 0.001), faecal consistency (CC = −0.13, *p* = 0.002) and age (CC = −0.13, *p* = 0.003) were observed. Age was significantly but weakly correlated with lactation (CC = −0.13, *p* = −0.003), BCS (CC = −0.11, *p* = 0.008), coat condition (CC = −0.09, *p* = 0.03), lesions on the body (CC = 0.11, *p* = 0.007), faecal consistency (CC = 0.08, *p* = 0.04), teat and udder score (CC = 0.11, *p* = 0.007), ocular lesions (CC = 0.10, *p* = 0.02), hock joint swelling (CC = 0.13, *p* = 0.002), hock joint hairloss (CC = 0.15, *p* = 0.001) and hock joint ulceration (CC = 0.11, *p* = 0.01). In a study on dairy cows [29] greater hair cortisol concentrations were reported in heifers than two-year-old cows. This was explained because of the diffusion of circulating cortisol concentrations in blood into the hair follicles following the stimulation of the adrenal gland of the cows by the foetal pituitary adrenal axis. However, the pregnancy of these cows could be the confounding factor in this elevation of hair cortisol levels. Similar correlations between lameness and dirtiness, hock lesions and lactations have been observed in previous studies [83,84,85].

All of the locations reflecting dirtiness of the cows i.e., flanks, udder and/or hind limbs, had significant positive relationships with carpal joint injuries (CC = 0.32, *p* ≤ 0.001), claw overgrowth (CC = 0.27, *p* ≤ 0.001), lameness (CC = 0.27, *p* ≤ 0.001), nasal discharge (CC = 0.11, *p* = 0.01), diarrhoea (CC = 0.12, *p* = 0.004), lesions on the body (CC = 0.33, *p* ≤ 0.001) and skin tenting time (CC = 0.24, *p* ≤ 0.001). The interrelationships between these parameters of cleanliness and cow health suggest a cumulative stress on the cows which could have been revealed by the elevated hair cortisol concentrations. Similar univariable relationships have been observed between different health and resource-based welfare parameters in welfare assessment in dairy cows [54].

Many of these welfare parameters were weakly correlated with each other and associations are not strong. However, we did not ignore them because they represented different aspects of welfare. For this reason, we analysed them separately with each other though it had the disadvantage that spurious associations might appear significant as multiple analysis were performed. So, we caution against the over-interpretation of single statistically significant variables, as concluded by Regula et al. [54].

### 4.3. Hair Cortisol and Shelter-Based Measures

The positive relationship of the hair cortisol concentration and the percentage of dung in the lying area of the cows in the shelters is almost certainly linked to the effect observed on the dirtiness of the cows. Dung in the lying areas makes the cows dirtier and hence susceptible to diseases and infection, leading to stress [66,67].

The negative relationship between hair cortisol concentration and dry bulb temperature in the shelters in our study is hard to explain. The thermal comfort zone for cattle is between 5 and 25 °C [86] and in our study, the median dry bulb temperature recorded in the shelters was 29.5 °C, above the thermoneutral zone. Examination of the data suggests that there was elevated hair cortisol when the ambient temperature was higher or lower than this range. Plasma cortisol concentrations have been found to be inconsistently related to higher temperatures, with studies showing an increase [87,88,89], decrease [90,91] or no changes [92,93,94] in cattle.

The negative association of the hair cortisol concentration and the access to the yards of the shelters (CC = −0.32, *p* = 0.01) suggests benefits of greater ease of movement. There were significant relationships between hair cortisol concentrations and hock lesions, cleanliness levels of cows, claw overgrowth and lameness in the univariable analysis in this study (Table 3). Reviews on studies about the benefits of loose housing with yards have shown that there is a low incidence of lameness, hoof pathologies, hock injuries, uterine affections and cleanliness in cows with such facilities, leading to less stress and better welfare [95]. Cattle like spending time on concrete pads rather than the muddy wet soil of the yards where poor hygiene prevails and might lead to immunosuppression [96]. One study [79] found no changes in the circulating plasma cortisol levels in pasture-grazed cows and totally housed cows. Another study [16] found elevation in hair cortisol levels when cows were moved from housing to summer pastures, though the freedom from confinement and better nutrition could be confounders. The lower hair cortisol concentrations in our study in cows having access to yards and pastures point to long term effects on the welfare of the cows.

### 4.4. Limitations of the Study

The parameters measured in this study were assumed to be accurate reflections of what we wanted to measure, which may not always have been the case. For example, we do not know whether cortisol concentration in hair is linearly related to the welfare of the cattle. Measurement techniques were, we believe, the best available and informed by a full literature review, but again may have inherent inaccuracies that we were not aware of. For example, we assumed that hair cortisol was best measured from tail hairs, as suggested previously [27], and did not compare cortisol between or within sites. The repeatability and reliability of many of the measures used is not yet known and should be the subject of further study.

In terms of the number of animals sampled, to our knowledge, this is the largest study so far on the assessment of hair cortisol concentrations in cows. There are conflicting reports on other studies conducted on the hair cortisol concentration of cattle, a topic which needs further assessment, for example, cows of different hair colours [13,97], to produce guidelines that can be built into future studies. However, the relationships observed suggest that hair cortisol is a good matrix to assess stress levels and hence the welfare status of cattle in facilities from a historical perspective.

## 5. Conclusions

Hair cortisol concentrations in shelters cows were elevated by the dirtiness of the cows, swellings and injuries of the limbs and body, age lactation and dehydration in the cows in the shelters. A negative association was found in the hair cortisol concentration and hock joint swelling, rumen fill and body hair loss. Evidence of a weak relationship was found between the hair cortisol concentration of the cows and the dry bulb temperature depicting the low levels in zones of thermoneutrality. Shelters providing access to the yards and having clean lying areas had cows with lower hair cortisol levels. This study was an analysis of welfare issues in the cow shelters at only one point in time, but a longitudinal study of cows from the time at which they enter the shelter could add further information on stress responses.

## Figures and Tables

**Table 1:** Descriptive statistics of the resource-based welfare parameters in shelters (*n* = 54).

Parameter	Median	First Quartile (Q_1_)	Third Quartile (Q_3_)	Interquartile Range (IQR)
Cows/shed	70	47.8	137.3	89.5
Shed area/cow (m^2^)	2.73	1.56	3.62	2.06
% dung in the lying area of the shed	15.0	5.0	40.0	35.0
% dung in the passages of shed	10.0	5.0	42.5	37.5
Height of shed eaves (m)	3.80	2.99	5.34	2.35
Gradient of shed lying area	1.46	0.96	2.2	1.23
Gradient of shed passages	2.36	1.27	3.52	2.24
Coefficient of friction of shed flooring (CoF)	0.43	0.27	0.65	0.37
Shed Luminosity level (lux)	582	89	1036	946
Shed noise level (decibels)	27.7	21.3	37.2	15.83
Shed dry bulb reading (°C)	29.5	27.2	32.8	5.6
Shed wet bulb reading (%)	34.0	24.7	45.2	20.50
Number of water points in the shelter	1.0	1.0	2.0	1.0
Percent dung in the yard	20	10	40	30
Yard area/cow (m^2^)	5.9	3.6	21.5	17.9
Coefficient of friction of yard flooring (COF_yards_)	0.64	0.34	0.68	0.34
Gradient of the yard flooring (degrees)	1.51	1.13	2.43	1.30
Nose levels in the yard (decibel)	25.3	20.3	33.0	12.7
Number of trees in the yard	2.0	0.0	6.0	6.0
Provision of ad lib water in the yard	10	0.0	1.0	1.0
Availability of access to yards (h)	8.0	4.0	24.0	20.0
Frequency of feeding to the cows (times/day)	3.0	2.0	3.0	1.0
Quantity of fodder provided (kg)	17.5	13.0	20.0	7.0

**Table 2:** Distribution of different animal-based welfare parameters in 54 cow shelters (*n* = 540 cows).

Parameter	% Score					
	0	1	2	3	4	5
Dirty hind limbs score (Scale 0–3)	2.41	43.3	41.8	12.4	-	-
Dirty udder score (Scale 0–3)	21.6	42.7	28.3	7.2	-	-
Dirty flanks score (Scale 0–3)	22.2	39.6	31.8	6.3	-	-
Body hair loss score (Scale 0–3)	46.6	27.5	23.1	2.5	-	-
Hock joint swelling score (Scale 0–3)	38.3	33.5	26.7	1.5	-	-
Hock joint hair loss score (Scale 0–3)	71.8	22.4	5.1	0.5	-	-
Hock joint ulceration score (Scale 0–3)	83.7	13.1	2.9	0.1	-	-
Carpal joint injuries score (Scale 0–3)	44.0	32.7	22.5	0.5	-	-
Neck lesions score (Scale 1–4)	-	93.5	5	0.5	0.9	
Ocular lesions score (Scale 0–1)	90	10	-	-	-	-
Lesions on the body score (Scale 0–3)	41.2	32.2	24.2	2.3	-	-
Nasal discharge score (Scale 0–1)	88.1	11.8	-	-	-	-
Diarrhoea score (Scale 0–1)	96.3	3.7	-	-	-	-
Faecal consistency score (Scale 1–5)	-	0.5	4.63	35.9	57.4	1.4
Rumen Fill Score (Scale 1–5)	-	0.0	4.4	38.5	56.8	0.1
Lameness score (Scale 1–5)	-	85.7	9.0	3.3	1.8	-
Claw overgrowth score (Scale 0–3)	54.0	34.6	9.2	2.0	-	-
Teat score (Scale 0–5)	14.6	82.4	0.9	0.3	0.0	1.6
Ectoparasitism score (Scale 0–4)	0.1	56.3	29.8	13.5	0.1	-
Skin tenting time score (Scale 0–4)	90.9	5.7	2.5	0.7	-	-
Rising up difficulty score (Scale 1–5)	-	93.3	3.8	2.9	-	-
Avoidance Distance score (Scale 0–3)	72.0	20.3	5.8	1.9	-	-

**Table 3:** Spearman’s Rank Correlation coefficients for hair cortisol concentration (pg/mg) with other animal-based parameters, together with a *p*-value for each correlation.

Animal-Based Parameter	Correlation Coefficient	*p*-Value
Dirty hind limbs score	0.232	<0.001
Dirty udder score	0.270	<0.001
Dirty flanks score	0.297	<0.001
Hock joint hair loss score	0.086	0.046
Hock joint ulceration score	0.213	<0.001
Carpal joint injuries score	0.276	<0.001
Diarrhoea score	0.152	<0.001
Rumen fill score	−0.224	<0.001
Claw overgrowth score	0.157	<0.001
Lameness score	0.177	<0.001
Lesions on the body score	0.176	<0.001
Avoidance distance score	0.222	<0.001
Age	0.111	0.012
Rising up difficulty score	0.270	<0.001
Lactation	−0.090	0.041
Body Condition Score (BCS)	−0.173	<0.001
Ocular lesions score	0.100	0.023
Nasal discharge score	0.149	0.001
Teat and udder score	0.169	<0.001

**Table 4:** Spearman’s Rank Correlation coefficients with *p*-values for hair cortisol concentration (pg/mg) with resource-based parameters.

Resource-Based Parameter	Correlation Coefficient	*p*-Value
Shed runoff in the lying area	0.298	0.028
Availability of access to yards	−0.370	0.006
Cleaning of the areas in addition to sheds and yards	−0.317	0.019

**Table 5:** Spearman’s Rank Correlation coefficients with *p*-values for hair cortisol concentration (pg/mg) with animal-based and resource-based parameters which were not significant (*p* > 0.05).

Parameter	Correlation Coefficient	*p*-Value
Temperament score	−0.029	0.511
Hock joint swelling score	0.066	0.137
Neck lesions score	0.012	0.788
Hampered respiration score	−0.066	0.136
Diarrhoea score	0.040	0.366
Vulvar discharge score	0.056	0.209
Faecal consistency score	−0.042	0.344
Ectoparasitism score	0.021	0.635
Shed flooring	−0.007	0.879
Shed bedding type	0.044	0.319
% dung in the lying area	−0.082	0.062
% dung in the passages	0.003	0.947
Presence of urine in shed passages	0.059	0.182
Thickness of bedding	0.044	0.316
Type of yard flooring	0.061	0.166
% dung in the yard	0.076	0.109
Area/cow in the shed	−0.056	0.207
Area/cow in the yard	−0.035	0.466
Frequency of scrapping the floors	−0.014	0.757
Method of floor scrapping	−0.024	0.594

**Table 6:** Regression analysis of animal-based parameters significantly related (*p* < 0.05) to hair cortisol concentration in log_10_pg/mg.

Parameter	Coefficient	SE of Coefficent	*p*-Value	VIF
Constant	0.20	0.084	0.017	
Dirty flanks	0.07	0.014	≤0.001	1.46
Body hair loss	−0.06	0.018	0.001	2.47
Hock joint ulceration	0.03	0.015	0.04	1.12
Carpal joint injuries	0.04	0.013	0.002	1.21
Rumen fill score	−0.06	0.019	0.002	1.17
Lesions on the body	0.03	0.018	0.04	2.39
Skin tenting time (s)	0.08	0.025	≤0.001	1.15
Age of cows (years)	0.005	0.002	0.03	1.09

VIF = Variance Inflation factor; SE = Standard Error.

**Table 7:** Regression analysis of resource-based parameters significantly (*p* < 0.05) related to hair cortisol concentration (log_10_pg/mg).

Parameter	Coefficient	SE of Coefficent	*p*-Value	VIF
Constant	6.15	0.881	≤0.001	
Dung in the lying area of shed (%)	0.01	0.005	0.02	1.83
Dry bulb temperature in the shed (°C)	−0.15	0.029	0.001	2.00
Duration of access to yards (h/day)	−0.07	0.024	0.015	1.16

VIF = Variance Inflation factor; SE = Standard Error.

**Table 8:** Comparative results of studies on the analysis of hair cortisol concentration in cattle.

Reference	Hair Cortisol Concentration (pg/mg)	Sample Size
Burnett et al. [18]	5.7 ± 1.7	18
del Rosario et al. [17]	12.15 ± 1.85	5
Moya et al. [27]	2.35 ± 0.176	12
Comin et al. [15]	2.1 ± 0.10–2.9 ± 0.17	83
Comin et al. [16]	3.29 (0.76–20.41) 5.12 (1.62–28.95)	257 218
Peric et al. [29]	Holsteins: 5.38 (1.91–27.95) Crossbreds: 4.40 (2.11–41.74)	142 148
Tallo-Parra et al. [13]	White hair: 2.1 ± 1.10 Black hair: 3.9 ± 1.44	17

## Supplementary Materials

The following are available online at https://www.mdpi.com/2076-2615/9/5/248/s1, Table S1: the data of this study.

## Appendix A

**Table A1:** Cow-based parameters used for the assessment of the welfare of cows in shelters.

Parameter	Description	Scales and Scores
Lactation		0—Non-lactating; 1—Lactating
Body Condition Score (BCS) [30,31]	A cow with a score of ≤1.25 was considered emaciated, 1.5–2 thin, 2.25–3.75 normal and 4 or more obese Visual examination	1 to 5 with increments of 0.25.
General temperament [32]	Visual examination	0—docile; 1—aggressive
Dirtiness of the hind limbs, udder and flanks [33]	By visual inspection of the cows from both sides (left and right) and from behind	1—no dirtiness; 2—mildly dirty (small soiled areas of dirtiness with no thick scabs); 3—medium dirtiness (large soiled areas but with <1 cm thick scabs of dung); 4—severely dirty (large soiled areas with >1 cm thick dung scabs);
Body hair loss [33]	Visual inspection	0—absence of hair loss;1—mild; 2—medium; 3—severe
Hock joint swellings [33,34]	Visual examination	0—no swollen joint; 1—mild swollen joint; 2—medium swollen joint; 3—severely swollen joint
Hock joint hair loss and ulceration [33,34]	Visual examination	0—no hair loss or ulceration; 1—mild hair loss or ulceration <2 cm^2^; 2—medium hair loss or ulceration (approximately 2.5 cm^2^); 3—severe hair loss or ulceration >2.5 cm^2^
Carpal joint injuries [34]	Visual examination	0—no skin change; 1—hairless; 2—swollen; 3—wound(s)
Lesions from shelter furniture [35]	Visual examination	0—normal (no lesions present); 1—small area of hair loss; 2—moderate area of hair loss and/or thickening of the skin; 3—severe (a large area of hair loss and/or breakage of the skin
Neck lesions [36]	Visual examination	1—no observable skin change; 2—hair loss; 3—swollen; 4—closed wounds (haematomas or closed abscesses); 5—open wounds
Ocular lesions [37]	Visual examination	0—absent; 1—present
Nasal discharge [37]	Visual examination	0—absent; 1—present
Hampered respiration [37]	Visual examination	0—absent; 1—present
Diarrhoea [37]	Visual examination	0—absent; 1—present
Vulvar discharge [37]	Visual examination	0—absent; 1—present
Rumen Fill Score [38]	Visually by standing behind the cow on the left side and observing the left paralumbar fossa between the last rib, the lumbar transverse processes and the hip bone	1—the paralumbar fossa is empty, presenting a rectangular cavity that is more than a hand’s width behind the last rib and a hand’s width under the lumbar transversal processes; 2—the paralumbar fossa forms a triangular cavity with a width about the size of a hand behind the last rib, but less than this under the lumbar transverse processes; 3—the paralumbar fossa forms a cavity less than a hand’s width behind the last rib and about a hand’s width vertically downwards from the lumbar transverse processes and then bulges out; 4—the paralumbar fossa skin covers the area behind the last rib and arches immediately outside below the lumbar transverse processes due to a bloated rumen; 5—the rumen is distended and almost fills up the paralumbar fossa; the last rib and the lumbar transverse processes are not visible
Faecal consistency [38]	Visual inspection	1—thin and watery and not truly recognizable as faeces; 2—thin custard-like consistency, structurally recognizable as faeces, splashing out wide upon falling on the floor; 3—thick custard-like consistency, making a plopping sound while falling on the floor and a well-circumscribed pad which spreads out and is about 2 cm thick; 4—stiff with a heavy plopping sound while falling on the floor and a proper circumscribed pad with visible rings and minimal spreading out; 5—hard faecal balls like horse faeces
Lameness Score [39]	1 to 5 scale Visual examination	1—not lame (smooth and fluid movement) 2—mildly lame but not observable easily (an imperfect gait but able to freely move with a mildly arched back) 3—moderately lame (able to move but not freely, with an arched back) 4—lame, with inability to move freely with and asymmetrical gait and abnormal head movement 5—severely lame (severely restricted in movement, requiring considerable encouragement to move, and a severely arched back)
Claw overgrowth [35]	Visual examination	0—Normal claws; 1—Mild claw overgrowth; 2—Moderate claw overgrowth; 3—Severe claw overgrowth
Skin lesions/Integument alterations [40]	Visual examination	0—normal (no apparent lesions); 1—mild hair loss (<2 cm^2^); 2—moderate (>2 cm^2^ hair loss and inflamed skin); 3—severe (a large >4 cm^2^ area of hair loss with extensive skin inflammation and breakage)
Oral lesions	Visual examination	0—absent; 1—present
Teat and/or udder condition	Visual inspection	1—Lactating udder and teats; 2—Non-lactating udder and teats; 3—Teat cracks; 4—Warts on teats and udder; 5—Acute lesions on the teats and udder; 6—Chronic lesions on teats and udder
Skin tenting time [69]	Visual examination by skin pinch of the cervical region of neck	1—≤2 s; 2—>2 s; 3—≥6 s
Ectoparasitism [41]	Visual examination	1—Absence of ectoparasites; 2—Mild infestation—no lesions (not easily visible by naked eye but on tactile perception in the neck region; 3—Moderate—mild infestation visually observable ectoparasites or immature forms or eggs in the neck, groin, peri rectal, tail root and switch regions; 4—Severe—observation of mature ectoparasites all over the body especially regions mentioned in score 3
Avoidance Distance (AD) [42]	Cows that were standing at the feeding manger were approached at the front at a rate of one step per second, starting at 2 m from the manger. The distance between the assessor’s hand and the cow’s head was estimated at the moment the cow moved away and turned its head.	0—touched; 1—0 to 50 cm; 2—51 to 100 cm; 3—>100 cm
Rising difficulty [43,44]	All cows lying in the shelter were coaxed to get up with use of a minimum amount of force. If the presence of the assessor did not evoke rising they were given one or two gentle slaps on the back, followed by a break of 5 s, then more slaps with slightly more force if required, up to a maximum of 30 s.	1—Normal (smooth and a normal sequence of rising behaviour; 2—Easy but slightly interfered (smooth movement with slight twisting of the head but with normal sequence of rising process; 3—Uneasy with effort (sudden movement and difficulty in rising with awkward twisting of the head and neck but following a normal sequential rising process); 4—Abnormal (uncharacteristic sequence of a rising event); 5—refused to get up

**Table A2:** Univariate correlations between variables in the animal-based measures.

	Cortisol	Lactation	BCS	Temperament	Dirty Hind Limbs	Dirty Udder	Dirty Flanks
Lactation	−0.090						
	0.041						
BCS	−0.173	0.150					
	0.000	0.001					
Temperament	−0.029	0.072	0.279				
	0.511	0.104	0.000				
Dirty Hind Limbs	0.232	0.049	−0.166	−0.067			
	0.000	0.269	0.000	0.132			
Dirty Udder	0.270	0.027	−0.255	−0.057	0.765		
	0.000	0.544	0.000	0.195	0.000		
Dirty Flanks	0.297	0.047	−0.236	−0.061	0.747	0.906	
	0.000	0.291	0.000	0.170	0.000	0.000	
Body Hair Loss	0.083	−0.068	−0.453	−0.112	0.263	0.423	0.421
	0.060	0.124	0.000	0.011	0.000	0.000	0.000
Hock Joint Swell	0.066	−0.058	0.038	−0.004	0.146	0.155	0.143
	0.137	0.188	0.396	0.920	0.001	0.000	0.001
Hock Joint Hair Loss	0.155	−0.077	−0.207	−0.022	0.040	0.101	0.093
	0.000	0.083	0.000	0.618	0.365	0.022	0.035
Hock Joint Ulcer	0.213	−0.018	−0.035	0.032	0.043	0.106	0.125
	0.000	0.677	0.432	0.469	0.329	0.016	0.005
Carpal Joint Injuries	0.276	0.009	−0.060	0.053	0.200	0.323	0.335
	0.000	0.847	0.174	0.233	0.000	0.000	0.000
Neck Lesions	0.012	−0.007	−0.150	−0.038	0.059	0.049	0.046
	0.788	0.871	0.001	0.395	0.186	0.267	0.296
Ocular Lesions	0.100	−0.035	−0.067	−0.019	0.007	0.069	0.065
	0.023	0.435	0.131	0.666	0.872	0.121	0.144
Coughing	0.060	−0.017	−0.071	−0.027	0.074	0.076	0.076
	0.175	0.698	0.110	0.538	0.097	0.086	0.085
Nasal Discharge	0.149	−0.080	−0.114	−0.048	0.113	0.114	0.084
	0.001	0.069	0.010	0.279	0.010	0.010	0.057
Hampered Respiration	−0.066	−0.017	−0.010	−0.027	−0.043	−0.011	−0.012
	0.136	0.698	0.817	0.538	0.328	0.796	0.786
Diarrhoea	0.040	0.001	−0.028	0.074	0.115	0.126	0.140
	0.366	0.977	0.520	0.093	0.009	0.004	0.002
Vulvar Discharge	0.056	0.006	−0.006	−0.050	0.052	0.069	0.091
	0.209	0.893	0.887	0.261	0.236	0.120	0.040
Rumen Fill Score	−0.224	0.016	0.182	0.016	−0.054	−0.168	−0.224
	0.000	0.714	0.000	0.725	0.225	0.000	0.000
Lameness	0.177	−0.010	−0.105	−0.065	0.205	0.257	0.255
	0.000	0.820	0.017	0.144	0.000	0.000	0.000
Lesions on the Body	0.176	−0.048	−0.375	−0.108	0.166	0.313	0.334
	0.000	0.276	0.000	0.015	0.000	0.000	0.000
Claw Overgrowth	0.157	0.052	−0.087	0.005	0.175	0.273	0.277
	0.000	0.238	0.049	0.914	0.000	0.000	0.000
Faecal Consistency	−0.042	−0.136	−0.135	−0.124	−0.209	−0.175	−0.194
	0.344	0.002	0.002	0.005	0.000	0.000	0.000
Coat Condition	−0.049	0.129	0.617	0.232	−0.265	−0.367	−0.354
	0.265	0.004	0.000	0.000	0.000	0.000	0.000
Teat Score	0.169	−0.596	−0.228	−0.100	0.013	0.041	0.037
	0.000	0.000	0.000	0.024	0.770	0.359	0.404
Ectoparasitism	0.021	−0.149	−0.203	0.033	0.149	0.249	0.220
	0.635	0.001	0.000	0.454	0.001	0.000	0.000
Skin Tenting Time	0.178	−0.053	−0.206	−0.057	0.173	0.245	0.259
	0.000	0.234	0.000	0.196	0.000	0.000	0.000
Age	0.111	−0.132	−0.117	−0.061	−0.012	0.029	−0.000
	0.012	0.003	0.008	0.166	0.783	0.511	1.000
	**Body Hair Loss**	**Hock Joint Swell**	**Hock Joint Hair Loss**	**Hock Joint Ulcer**	**Carpal Joint Injuries**	**Neck Lesions**	**Ocular Lesions**
Hock Joint Swell	0.056						
	0.207						
Hock Joint Hair Loss	0.162	0.381					
	0.000	0.000					
Hock Joint Ulcer	0.051	0.267	0.611				
	0.251	0.000	0.000				
Carpal Joint Injuries	0.112	0.198	0.172	0.247			
	0.012	0.000	0.000	0.000			
Neck Lesions	0.185	−0.106	−0.006	−0.031	0.048		
	0.000	0.016	0.893	0.487	0.278		
Ocular lesions	−0.005	0.048	0.073	0.079	0.039	−0.034	
	0.904	0.278	0.101	0.075	0.383	0.436	
Coughing	0.080	0.026	0.057	0.073	0.019	−0.012	−0.015
	0.071	0.562	0.200	0.098	0.676	0.793	0.737
Nasal Discharge	0.091	0.029	0.131	0.092	0.129	0.009	0.048
	0.039	0.506	0.003	0.037	0.003	0.847	0.279
Hampered respiration	−0.044	−0.055	−0.064	−0.041	−0.045	−0.012	0.132
	0.317	0.210	0.151	0.350	0.307	0.793	0.003
Diarrhoea	−0.003	−0.061	−0.018	0.002	0.072	−0.046	0.095
	0.949	0.166	0.679	0.960	0.106	0.303	0.032
Vulvar Discharge	0.049	−0.013	−0.034	−0.024	0.025	0.052	0.102
	0.273	0.778	0.438	0.590	0.565	0.239	0.021
Rumen Fill Score	−0.197	0.047	−0.000	−0.159	−0.147	−0.039	−0.183
	0.000	0.287	0.994	0.000	0.001	0.373	0.000
Lameness	0.091	0.080	0.054	0.095	0.201	0.034	0.051
	0.039	0.070	0.225	0.032	0.000	0.439	0.253
Lesions on the Body	0.708	0.021	0.165	0.150	0.110	0.173	0.067
	0.000	0.631	0.000	0.001	0.013	0.000	0.129
Claw Overgrowth	0.163	0.098	−0.004	0.081	0.252	0.054	0.131
	0.000	0.027	0.925	0.068	0.000	0.223	0.003
Faecal Consistency	0.023	0.003	0.087	0.061	−0.130	0.023	0.011
	0.609	0.938	0.050	0.168	0.003	0.608	0.797
Coat Condition	−0.494	−0.063	−0.138	−0.007	−0.098	−0.144	−0.001
	0.000	0.155	0.002	0.870	0.027	0.001	0.984
Teat Score	0.078	0.038	0.089	0.090	0.056	0.018	0.070
	0.076	0.397	0.045	0.041	0.204	0.684	0.113
Ectoparasitism	0.245	0.050	0.105	0.097	0.147	0.043	0.053
	0.000	0.257	0.017	0.028	0.001	0.337	0.229
Skin Tenting Time	0.177	−0.014	0.065	0.106	0.145	0.067	0.091
	0.000	0.748	0.143	0.017	0.001	0.131	0.040
Age	0.155	0.135	0.150	0.112	0.143	0.046	0.100
	0.000	0.002	0.001	0.011	0.001	0.297	0.023
	**Coughing**	**Nasal Discharge**	**Hampered Respiration**	**Diarrhoea**	**Vulvar Discharge**	**Rumen Fill Score**	**Lameness**
Nasal Discharge	0.126						
	0.004						
Hampered Respiration	−0.002	−0.016					
	0.965	0.726					
Diarrhoea	−0.008	0.088	0.255				
	0.862	0.048	0.000				
Vulvar Discharge	0.264	0.095	0.264	0.042			
	0.000	0.032	0.000	0.344			
Rumen Fill Score	0.036	−0.022	0.036	−0.121	−0.068		
	0.412	0.625	0.412	0.006	0.122		
Lameness	0.105	0.094	−0.018	−0.005	0.070	−0.121	
	0.018	0.033	0.692	0.910	0.114	0.006	
Lesions on the Body	0.058	0.090	0.012	0.032	0.090	−0.323	0.125
	0.190	0.041	0.787	0.464	0.042	0.000	0.004
Claw Overgrowth	0.037	0.063	0.037	0.105	0.048	−0.187	0.346
	0.401	0.152	0.401	0.017	0.283	0.000	0.000
Faecal Consistency	0.034	−0.068	−0.050	−0.280	0.036	−0.000	−0.019
	0.449	0.126	0.258	0.000	0.410	0.996	0.665
Coat Condition	−0.046	−0.139	0.042	−0.019	0.017	0.099	−0.098
	0.299	0.002	0.339	0.673	0.701	0.026	0.027
Teat Score	0.014	0.074	0.014	−0.034	0.019	−0.069	0.040
	0.749	0.092	0.749	0.444	0.663	0.118	0.369
Ectoparasitism	0.076	−0.001	−0.037	0.055	0.097	0.004	0.028
	0.086	0.990	0.409	0.217	0.028	0.936	0.534
Skin Tenting Time	0.160	0.105	−0.013	0.122	0.173	−0.164	0.139
	0.000	0.017	0.765	0.006	0.000	0.000	0.002
Age	0.048	0.072	0.048	−0.009	0.058	−0.070	0.024
	0.281	0.103	0.281	0.839	0.189	0.113	0.590
	**Lesions on the Body**	**Claw Overgrowth**	**Faecal Consistency**	**Coat Condition**	**Teat Score**	**Ectoparasitism**	**Skin Tenting Time**
Claw Overgrowth	0.277						
	0.000						
Faecal Consistency	0.015	−0.069					
	0.733	0.121					
Coat Condition	−0.401	−0.092	−0.074				
	0.000	0.037	0.095				
Teat Score	0.107	−0.010	0.155	−0.213			
	0.015	0.820	0.000	0.000			
Ectoparasitism	0.225	0.132	0.061	−0.328	0.196		
	0.000	0.003	0.171	0.000	0.000		
Skin Tenting Time	0.252	0.193	0.085	−0.199	0.142	0.249	
	0.000	0.000	0.055	0.000	0.001	0.000	
Age	0.118	0.079	0.089	−0.095	0.118	0.025	−0.022
	0.007	0.075	0.045	0.031	0.007	0.579	0.616

**Table A3:** Univariate correlations between variables in the resource-based measures.

	Cortisol	Shed Flooring	Shed Bed Type	Shed %Dung LyA	Shed %Dung Pa	Shed Urine LA	Shed Urine in Pa
Shed flooring	−0.007						
	0.879						
Shed bed type	0.044	−0.284					
	0.319	0.000					
Shed %Dung LyA	−0.082	−0.198	0.044				
	0.062	0.000	0.315				
Shed %Dung Pa	0.003	−0.193	0.072	0.914			
	0.947	0.000	0.103	0.000			
Shed urine LA	0.105	0.179	−0.089	0.115	0.101		
	0.017	0.000	0.045	0.009	0.022		
Shed urine in Pa	0.059	0.183	−0.089	0.102	0.123	0.578	
	0.182	0.000	0.043	0.021	0.005	0.000	
Shed cleaned /cl	−0.095	0.169	−0.046	−0.622	−0.594	0.144	0.039
	0.032	0.000	0.298	0.000	0.000	0.001	0.373
Shed Bed Thickness	0.044	−0.284	1.000	0.050	0.077	−0.089	−0.089
	0.316	0.000	0.000	0.262	0.082	0.045	0.043
Shed run off Ly	0.196	0.206	−0.129	0.018	−0.015	−0.048	0.064
	0.000	0.000	0.003	0.687	0.731	0.274	0.145
Shed average gradient LyA	−0.109	−0.284	−0.010	0.057	0.058	−0.030	−0.124
	0.014	0.000	0.829	0.196	0.193	0.498	0.005
Shed Average gradient Pa	0.007	−0.320	0.221	0.139	0.132	−0.263	−0.087
	0.871	0.000	0.000	0.002	0.003	0.000	0.051
Yard flooring	0.061	0.264	−0.105	−0.021	0.057	0.016	0.081
	0.166	0.000	0.018	0.628	0.198	0.718	0.065
Yard %Dung	0.076	−0.360	0.218	0.285	0.384	−0.120	−0.293
	0.109	0.000	0.000	0.000	0.000	0.012	0.000
Yard urine	0.103	0.062	−0.072	−0.089	−0.064	0.478	0.324
	0.019	0.158	0.103	0.045	0.151	0.000	0.000
Yard area/cow	−0.035	−0.189	−0.029	0.018	0.019	−0.128	−0.315
	0.466	0.000	0.543	0.706	0.697	0.007	0.000
Yard avg gradient	−0.059	0.065	−0.094	0.008	−0.011	0.108	0.095
	0.231	0.188	0.055	0.874	0.828	0.027	0.054
Scraping Frequency	−0.014	0.466	−0.167	−0.405	−0.431	0.075	0.117
	0.757	0.000	0.000	0.000	0.000	0.092	0.008
Scraping method	−0.024	−0.018	−0.073	0.172	0.121	0.157	0.329
	0.594	0.685	0.101	0.000	0.006	0.000	0.000
Avoidance Distance	0.087	0.015	−0.125	−0.103	−0.074	0.231	0.024
	0.048	0.734	0.005	0.019	0.092	0.000	0.593
Rising up difficulty	0.227	0.091	−0.054	−0.005	−0.060	0.179	0.177
	0.000	0.040	0.225	0.914	0.172	0.000	0.000
Shed area/cow	−0.056	−0.040	−0.038	−0.032	−0.097	−0.097	0.044
	0.207	0.366	0.388	0.473	0.028	0.028	0.319
	**Shed Cleaned/cl**	**Shed Bed Thickness**	**Shed Run off LyA**	**Shed Average Gradient LyA**	**Shed Average Gradient Pa**	**Yard Flooring**	**Yard %Dung**
Shed Bed Thickness	−0.050						
	0.258						
Shed run off Ly	−0.107	−0.129					
	0.016	0.003					
Shed average gradient	−0.061	−0.009	−0.036				
	0.166	0.832	0.422				
Shed Average gradient	−0.186	0.223	0.066	0.287			
	0.000	0.000	0.142	0.000			
Yard flooring	0.112	−0.105	−0.140	−0.072	−0.067		
	0.011	0.018	0.001	0.103	0.135		
Yard %Dung	−0.424	0.220	0.012	0.219	0.252	−0.301	
	0.000	0.000	0.809	0.000	0.000	0.000	
Yard urine	0.171	−0.072	−0.093	0.148	−0.075	0.305	−0.112
	0.000	0.103	0.035	0.001	0.094	0.000	0.018
Yard area/cow	0.071	−0.023	0.265	0.165	0.152	−0.420	0.318
	0.134	0.634	0.000	0.001	0.001	0.000	0.000
Yard avg gradient	0.092	−0.092	−0.234	0.298	0.030	−0.059	−0.112
	0.061	0.062	0.000	0.000	0.548	0.233	0.022
Scraping Frequency	0.407	−0.167	0.051	−0.311	−0.355	0.072	−0.530
	0.000	0.000	0.251	0.000	0.000	0.102	0.000
Scraping method	−0.187	−0.073	0.038	−0.193	0.090	0.023	−0.169
	0.000	0.101	0.394	0.000	0.044	0.598	0.000
Avoidance Distance	−0.047	−0.125	0.246	0.063	−0.035	−0.166	−0.070
	0.286	0.005	0.000	0.156	0.442	0.000	0.140
Rising up difficulty	−0.095	−0.054	0.176	−0.029	−0.128	−0.132	−0.103
	0.032	0.225	0.000	0.520	0.004	0.003	0.031
Shed area/cow	0.068	−0.034	0.137	−0.213	−0.035	−0.169	−0.038
	0.125	0.440	0.002	0.000	0.436	0.000	0.423
	**Yard Urine**	**Yard Area/Cow**	**Yard Avg Gradient**	**Scraping Frequency**	**Scraping Method**	**Avoidance Distance**	**Rising up Difficulty**
Yard area/cow	−0.117						
	0.014						
Yard avg gradient	0.064	−0.097					
	0.191	0.049					
Scraping Frequency	−0.074	−0.036	−0.020				
	0.097	0.453	0.689				
Scraping method	0.238	−0.126	−0.032	−0.249			
	0.000	0.008	0.513	0.000			
Avoidance Distance	0.302	0.111	0.029	−0.165	0.192		
	0.000	0.019	0.553	0.000	0.000		
Rising up difficulty	0.121	−0.167	−0.024	0.026	0.106	0.297	
	0.006	0.000	0.632	0.557	0.017	0.000	
Shed area/cow	−0.149	0.065	−0.094	0.167	0.069	−0.311	−0.009
	0.001	0.174	0.056	0.000	0.120	0.000	0.844
